# Microbial colonisation of the neonatal beef calf gastrointestinal tract: limited influence of birth delivery method across five anatomical regions

**DOI:** 10.3389/fmicb.2026.1883207

**Published:** 2026-07-29

**Authors:** Michelle M. Stafford, Paul E. Smith, Sinead M. Waters, Frank Buckley, Stuart F. Kirwan, Eoin O’Hara, David A. Kenny

**Affiliations:** 1 Department of Teagasc Animal and Bioscience Research, Meath, Ireland; 2 School of Biological, Earth and Environmental Sciences, University College Cork, Cork, Ireland

**Keywords:** 16S rRNA, colonisation, delivery method, gastrointestinal tract, microbiome, neonatal calf

## Abstract

The microbiome assembly of the neonatal gastrointestinal tract is critical for immune and metabolic development, and birth delivery method can influence initial microbial colonisation. Using 16S rRNA gene amplicon sequencing, we investigated the effect of transvaginal and elective caesarean section delivery on microbial composition across five gastrointestinal regions (colon, ileum, jejunum, meconium, and rumen) in Aberdeen Angus calves (transvaginal, TV; *n =* 12; caesarean section, CS; *n =* 10) across two research farms. Absolute microbial quantities were measured using 16S rRNA qPCR. Alpha diversity (Observed, Shannon, Simpson) was compared between delivery methods within each region using Wilcoxon rank-sum tests, whereas beta diversity (Bray–Curtis PERMANOVA) and differential abundance (MaAsLin2) analyses were conducted with sex and farm as fixed effects. Alpha diversity varied among gastrointestinal regions (*p* < 0.05) but did not differ between delivery methods within any region (all BH-adjusted q ≥ 0.74). Gastrointestinal region was the primary determinant of microbial community composition (R^2^ = 0.24, *p* < 0.001), with delivery method explaining minimal additional variance. A statistically significant but weak Region × Delivery interaction was observed (R^2^ = 0.06, *p* = 0.02), indicating that any influence of delivery method was site-dependent rather than uniform across the gastrointestinal tract. Farm explained negligible variation (R^2^ = 0.01, *p* = 0.17) and no overall delivery method effect was detected. Per-region analyses found no statistically significant treatment effects within individual regions (all *p* ≥ 0.06), suggesting the interaction reflected modest region-specific trends rather than strong site-specific shifts. Microbial load and community composition were comparable between CS and TV calves at birth. Early microbial establishment was therefore primarily driven by anatomical site, with postnatal environmental exposure and host–microbe interactions likely playing a greater role than delivery method in shaping early gastrointestinal microbial assembly in neonatal beef calves.

## 1 Introduction

Colonisation of the mammalian neonatal gastrointestinal tract (GIT) has traditionally been considered to begin at birth following passage through the birth canal and subsequent external environment (Taschuk and Griebel, 2012). Notwithstanding this, bacterial DNA and microbial communities have been detected in the foetal GIT and associated fluids during gestation (Guzman et al., 2020; Rackaityte et al., 2020) challenging the sterile-foetus paradigm. These findings have remained contentious due to concerns regarding contamination and limited evidence of immune activation (de Goffau et al., 2019; Walter and Hornef, 2021). A much more accepted theory is microbial inoculation occurring during passage through the birth canal, with vertical transmission of maternal microbiota playing a critical role in shaping early microbial establishment (Alipour et al., 2018; Klein-Jöbstl et al., 2019).

These early microbial populations play an important role in the development of a functional intestinal barrier, stimulate immune maturation, and support the overall health and growth of neonatal calves (Alipour et al., 2018; Klein-Jöbstl et al., 2019; Zhu et al., 2021). In ruminants, production performance and feed efficiency has been linked to early microbial establishment (Morgavi et al., 2020; Amin and Seifert, 2021; Arshad et al., 2021). Although early life microbial succession in ruminants has been explored, particularly within the rumen, microbial colonisation of the neonatal lower gastrointestinal tract at birth remains poorly characterised. Specifically, no study has simultaneously examined how delivery method and anatomical niche ecology interact to shape early microbial communities across multiple intestinal regions in bovine neonates, particularly in low-biomass tissues where contamination represents a substantial interpretive challenge.

Transvaginal (TV) birth facilitates the initial exposure of the neonate to the dam’s vaginal, faecal, and skin microbiota, as well as to microbes in colostrum and the environment (Biasucci et al., 2008; Clemmons et al., 2017; Kalbermatter et al., 2021). This exposure provides initial microbial inoculum that seeds the calf’s skin, oral cavity and GIT. In humans, where the mechanistic link between vaginal delivery and neonatal microbiome seeding has been most extensively characterised, vaginally delivered neonates harbour microbiota closely resembling their mother’s vaginal community, with enrichment of taxa such as *B. longum*, and *B. catenulatum*, Lactobacillus, Prevotella, and Sneathia species (Biasucci et al., 2008; Houghteling and Walker, 2015; Zhang et al., 2021b). While the specific taxa differ between species, analogous maternal-to-neonatal microbial transfer during vaginal delivery has been reported in cattle, with neonatal calf faecal microbiota more closely resembling the dam’s vaginal microbiota than other maternal sites (Clemmons et al., 2017; Klein-Jöbstl et al., 2019).

Although maternal microbial exposure during birth may introduce early colonising taxa, these signals are typically low in biomass and short-lived, raising the possibility that anatomical site-specific ecological factors exert a stronger influence on initial microbial establishment than delivery pathway alone (Yeoman et al., 2018). Initial colonisers of the GIT have been demonstrated to utilise available oxygen and create an anaerobic environment suitable for strict anaerobic colonisers such as *Bifidobacterium* and *Bacteroides*, which have been associated with reduced allergy incidence, short-chain fatty acid production, and enhanced immune tolerance in human neonates (Malmuthuge et al., 2015; Zhang et al., 2021b).

Early life microbial communities are critical for immune tolerance and disease resistance. Calves with neonatal diarrhoea (Jessop et al., 2024) and pneumonia (Wang et al., 2025) typically have a lower bacterial diversity than healthy calves. *Faecalibacterium* and *Bifidobacterium* (Zhang et al., 2021b) play an important role in suppressing enteropathogenic colonisation and are negatively correlated with diarrhoea incidence in neonatal ruminants. Conversely, genera enriched in dysbiotic states, including *Enterococcus, Ligilactobacillus*, *Lactobacillus*, *Gallibacterium*, *Streptococcus*, and *Escherichia*/*Shigella*, have been identified at elevated abundances in calves with enteric disease (Ma et al., 2020; Slanzon et al., 2022; Du et al., 2023). These compositional shifts highlight the importance of a balanced microbial composition for calf performance and health.

In humans, neonates delivered by caesarean section (CS) bypass exposure to maternal vaginal and intestinal microbiota, instead acquiring microbes primarily from skin and the surrounding environment (Inchingolo et al., 2024). This has been associated with reduced microbial diversity and enrichment of opportunistic taxa such as *Staphylococcus* and *Corynebacterium* in human neonates *(Biasucci et al.,**2008*; *Long et al.,**2021**).* While delivery method is recognised as an important determinant of early microbial colonisation in humans, its influence on the GIT colonisation in cattle remains poorly characterised.

There has been considerable debate regarding whether microbial colonisation begins in utero. While bacterial DNA has been detected in some placental and foetal samples (Guzman et al., 2020), studies employing rigorous contamination controls have found little evidence of a distinct placental microbiome, and the absence of prenatal colonisation in germ-free animal models supports postnatal environmental exposure as the primary driver of GIT establishment (Lauder et al., 2016; de Goffau et al., 2019; Walter and Hornef, 2021). Given the low-biomass nature of neonatal gastrointestinal tissues at birth, rigorous negative controls and contamination-aware bioinformatic approaches are essential for accurate characterisation of these communities and were a central consideration in the design of the present study.

Given the limited characterisation of delivery method effects on GIT microbial colonisation in bovine neonates, the aim of this study was to investigate the influence of delivery method (TV vs. CS) on early microbial colonisation across five gastrointestinal regions in neonatal calves, including the rumen, ileum, jejunum, and colon, as well as unvoided meconium, using 16S rRNA gene amplicon sequencing and qPCR to assess differences in microbial diversity and community composition between delivery modes. The working hypothesis was that delivery-method effects would be most apparent in the proximal regions of the sampled GIT, specifically the rumen and jejunum, which are amongst those exposed earliest and most directly to maternal vaginal and environmental microbiota via oral ingestion and subsequent luminal transit during and immediately after parturition, whereas the more distal ileum, colon, and meconium were expected to show comparatively limited delivery-associated differences at birth.

## 2 Materials and methods

### 2.1 Ethical statement

The experiments described in this trial were conducted with the prior approval of the Teagasc Animal Ethics Committee (TAEC) and the Irish Health Products Regulatory Association (HPRA; Project Authorisation AE19132/P025). A full breakdown of the sample sizes by replicate, farm, and calf gender is provided in Supplementary Table S1. Sample size calculations were based on an anticipated difference of 30% between delivery method groups in rumen microbial diversity, with a coefficient of variation of 20% derived from previous published studies and in-house data, with *α* = 0.05, and power of 80%.

### 2.2 Experimental design, animal trial & sample collection

The management of the animals included in this trial has been described previously (Surlis et al., 2017; O'Hara et al., 2020; Stafford et al., 2025). Aberdeen Angus Heifers (*n =* 93) were purchased and housed at the Teagasc Mellows Campus, Athenry, Co. Galway, Ireland (Farm 1; F1). Oestrus synchronisation of the heifers was conducted prior to being served with artificial insemination from a single Aberdeen Angus bull sire, resulting in 67 viable pregnancies. This cohort underpinned a wider experimental programme following calves across multiple postnatal timepoints, with rumen and colon microbial ontogeny at these timepoints reported separately (Lyons et al., 2020; O'Hara et al., 2020; Stafford et al., 2025). The present study reports on the complete Day 0 (birth) cohort from this design, 22 heifers in total, randomly assigned to caesarean section (CS, *n =* 10) or transvaginal delivery (TV, *n =* 12). No further selection or exclusion criteria were applied within this cohort. The heifers assigned to replicate 1 and 2 were housed in F1 (*n =* 11) and the heifers assigned to replicate 3 and 4 (*n =* 11) were transported in the third trimester to the Department of Agriculture, Food and Marine (DAFM) Longtown Research Facility in Clane, Co. Kildare, Ireland (Farm 2; F2) prior to calving. The transport duration lasted 2 h, with every measure taken to reduce stress on the animals. The heifers in F2 were housed with feeding practices similar to those at F1. Treatment groups were balanced across farms, with Farm 1 (Replicates 1 and 2) comprising six CS and six TV calves, and Farm 2 (Replicates 3 and 4) comprising four CS and six TV calves. A minor treatment imbalance at Farm 2 arose from logistical constraints during data collection; farm was therefore included as a fixed covariate in the combined PERMANOVA model to account for any residual farm-specific environmental variation.

The heifers were housed indoors for 8 weeks prior to their expected calving date with *ad libitum* access to moderate energy density grass silage and 2 kg concentrate daily. A week before the expected calving the heifers were randomly assigned to either CS or TV treatment groups, balanced by sex and sample size. Heifers were assigned to the following group: CS (*n =* 10) or TV (*n =* 12). To ensure that calvings were adequately supervised and managed, parturition was induced for the heifers in the TV group in order to enter labour approximately 48 h before their projected calving date, by administering 2 mL of a prostaglandin F2_α_ analogue (Estrumate™, Merck, NJ, USA). The elective caesarean sections for the CS group were performed by an experienced veterinary surgeon using standard protocols. Heifers were administered with local anaesthetic (lidocaine) in accordance with normal veterinary procedures. Calves from both CS and TV were delivered onto plastic sheets to minimise their contact with the environment and had no contact with the dam.

The calves selected for this study were euthanised via a single, rapid and humane lethal intravenous injection of pentobarbital sodium within 10 min of their birth. There was no prior sedation or analgesia administered before euthanasia, consistent with AVMA and EU guidelines for livestock. Efforts were made to minimise the stress of handling before euthanasia. The lack of a corneal reflex and the absence of cardiac activity was used to determine death, following this samples were collected within 25 min of the calf’s birth. The sampling was conducted in a dedicated surgery in both locations. The room was cleaned before sampling commenced and the area was thoroughly cleaned between the processing of each animal. Each calf was sampled in isolation using sterile single-use instruments. All surfaces and sampling equipment were treated with boiling water, ethanol, and RNase inhibitor prior to processing samples from each calf, consistent with previously published protocols from this animal model (Lyons et al., 2020; O'Hara et al., 2020). Sterile pre-labelled tubes were used to store all samples, which were immediately snap-frozen in liquid nitrogen.

The samples were collected separately, placed in sterile tubes, and snap frozen in liquid nitrogen, and then stored at -80 °C pending molecular analysis. Samples collected included colon content (CS *n =* 9 and TV *n =* 7), ileum content (CS *n =* 9 and TV *n =* 10), jejunum content (CS *n =* 9 and TV *n =* 7), meconium (CS *n =* 9 and TV *n =* 9) and rumen swabs (CS *n =* 9 and TV *n =* 10). Minor variation in the final sample numbers across the regions occurred due to sample loss at collection or during molecular analysis.

### 2.3 DNA extraction and sequencing

The samples were ground down under liquid nitrogen, using a pestle and mortar to a fine powder (Smith et al., 2020b; Scully et al., 2024; Stafford et al., 2025). A modified DNA purification protocol by Stevenson and Weimer (2007) method was used. In summary, 0.5 g of the powdered samples were added to a micro centrifuge tube containing 0.5 g of zirconium/silica beads (0.1 mm), 100 μL of 10% SDS, 700 μL of equilibrated phenol added and cell lysis performed through bead beating for 3 min at maximum speed. The samples were centrifuged at 12000 g for 5 min at room temperature. The liquid phase was transferred to a fresh tube, with a ~ 100 μL left between the aqueous solution and the interphase. The same method was followed for extraction with phenol: chloroform and chloroform, each step repeated and transferred to a fresh tube. Finally, 0.1 volume of 3 M sodium acetate and 0.6 volume isopropanol were added to the supernatant, and the reaction was left overnight. On the second day, the reaction was centrifuged at 16000 g for 30 min at 4 °C. The pellet was washed with cold 70% ethanol, centrifuged at 16000 g for 15 min at 4 °C and dried at 37 °C. The pellet was then re-suspended in 100 μL of TE (warmed to 50 °C) and incubated in a dry bath for 20 min at 55 °C. The rumen swab samples were placed in 750 μL PBS and vortexed for 1 min, with 240 μL of the resulting solution used for the extraction using the same method as previously described. DNA quantity was estimated using a NanoDrop 1,000 spectrophotometer. The quality of the concentrated DNA was assessed on 0.8% agarose gels before diluting the DNA to 100 ng/μl. DNA extractions were also performed on ZymoBIOMICS™ Microbial Community Standard (MC) (Zymo Research Corp., Irvine, CA, United States), included as a positive control to assess DNA extraction efficiency. The extractions on ZymoBIOMICS™ MC were subject to the same library preparation and sequencing regime as all other samples.

### 2.4 16S rRNA gene amplicon library preparation and sequencing

The DNA extractions were subject to 16S rRNA amplicon library preparation and sequencing completed by Macrogen (Seoul, South Korea) using the 515F/806R primers (Caporaso et al., 2011) targeting the V4 hypervariable region of the 16S rRNA gene designed with Nextera overhang adapters and Herculase II Fusion DNA Polymerase (Agilent, Santa Clara, CA, United States). The PCR conditions were as follows: initial denaturation at 95 °C for 3 min, followed by 25 cycles of denaturation at 95 °C for 30 s, annealing at 55 °C for 30 s, elongation for 72 °C for 30 s, final elongation at 72 °C for 5 min.

PCR amplicon purification was performed using the standard AMpure paramagnetic bead protocol (Beckman Coulter, Indianapolis, IN, United States). The amplicons underwent a second round of PCR to allow the attachment of dual indices and Illumina sequencing adapters using the Nextera XT indexing kit (Illumina, San Diego, CA, United States). The second round of PCR was performed under the following cycling conditions: initial denaturation at 95 °C for 3 min, followed by 10 cycles of 95 °C for 30 s, 55 °C for 30 s, and 72 °C for 30 s, with a final extension at 72 °C for 5 min. PCR products were subsequently purified using the AMPure paramagnetic bead protocol (Beckman Coulter, Indianapolis, IN, USA). Equimolar amounts of purified amplicons were pooled and sequenced on the Illumina MiSeq platform using the 500-cycle v2 reagent kit (Illumina, San Diego, CA, USA).

Quantitative PCR (qPCR) reactions were prepared in a final volume of 20 μL per well. The master mix consisted of 10 μL of Fast SYBR Green Master Mix with ROX (Applied Biosystems, Thermo Fisher Scientific), 1 μL of forward primer (515F), 1 μL of reverse primer (806R), and 7 μL of nuclease free water. DNA samples were diluted to a concentration of 5 ng/μL, and 1 μL of each diluted DNA sample was added directly to the bottom of each well in a 96 well plate. Subsequently, 19 μL of the master mix was added to each well using a multichannel pipette, mixed thoroughly, sealed with optical adhesive film, and centrifuged for 1 min. Reactions were run on the Applied Biosystems 7,500 Fast Real Time PCR System using the following conditions: experiment type was set to “Quantitation – Standard Curve” using SYBR Green chemistry, with a fast ramp speed (total run time approximately 40 min). Thermal cycling conditions were as follows: initial hold at 95 °C for 20 s, followed by 40 cycles of 95 °C for 3 s and 60 °C for 30 s. A melt curve analysis was performed immediately after amplification, consisting of 95 °C for 15 s, 60 °C for 1 min, 95 °C for 15 s, and a final step at 60 °C for 15 s. Targets and sample IDs were defined and mapped to their respective well positions using the instrument’s software prior to running the assay. qPCR was performed on all samples, including positive and negative controls, with the exception of the following due to insufficient DNA: colon (*n =* 2), ileum (*n =* 1), jejunum (*n =* 1), meconium (*n =* 1), and rumen swab (*n =* 1). Ct values were obtained for each sample alongside no template controls (H₂O), extraction negatives, and positive controls (Zymo DNA standard and/or *Staphylococcus aureus* DNA).

### 2.5 Bioinformatics analysis

Raw paired end reads (2 × 250 bp, V4 region; primers 515F/806R) were processed in R (v4.3.3) using the DADA2 pipeline (v1.30.0) (Callahan et al., 2016) with some minor modifications (Smith et al., 2020a). The quality of the forward and the reverse reads were evaluated by visually inspecting their Q scores confirming that the average quality score exceeded 30 for both forward and reverse reads. The forward and reverse reads were truncated at 200 bp and 180 bp, respectively, using the filterAndTrim() function to remove low quality bases, as quality profiles showed a marked decline beyond these positions. This truncation retained an expected overlap of ~60 bp for the V4 region (~250 bp), ensuring reliable merging of paired end reads. Reads were de-replicated and denoised using the dada() function, followed by merging of paired reads and removal of chimeric sequences.

Amplicon sequence variants (ASV) were inferred and compiled into a sequence table. Taxonomic classification was performed against the SILVA reference database (version 138.1), using a minimum bootstrap confidence threshold of 80 (via assignTaxonomy(minBoot = 80), consistent with Smith et al. (2020a). Sample metadata, taxonomic annotations, and ASVs were combined into a phyloseq object using the *phyloseq* package using version 1.46.0; (McMurdie and Holmes, 2013).

Potential contaminants were identified using the decontam package (v1.22.0) based on both prevalence (in negative controls) and frequency models (correlated with DNA concentration via qPCR Ct values). ASVs flagged by either method (union) were removed (*n =* 134, 0.77% of all ASVs detected), retaining 94.6% of reads. Rarefaction curves (Observed, Shannon) were generated pre- and post-decontamination with controls and then post-filtering to assess sequencing adequacy (Supplementary Figure S1). Alpha diversity metrics were calculated on data rarefied to the minimum sequencing depth retained across all samples after quality filtering and decontamination (1,612 reads per sample), consistent with even-depth rarefaction practice for low-biomass datasets (Weiss et al., 2017). A sensitivity analysis conducted at a higher rarefaction threshold of 5,000 reads per sample (*n =* 76 of 88 samples; ileum *n =* 11, reflecting the low-biomass nature of this region) confirmed the absence of significant delivery- method effects on alpha diversity across all regions and metrics (all BH-adjusted q = 1.00; Supplementary Table S5). Beta diversity and differential abundance analyses were performed on unrarefied data using compositional approaches, avoiding information loss associated with rarefaction for community-level comparisons. After decontamination, negative and positive controls were excluded; data were then split by domain (bacteria and archaea). For downstream analysis, we applied a ≥ 5% prevalence filter within each domain; for some summaries comparing TV vs. CS taxa were retained with relative abundance > 0.001 in ≥ 5% of samples. A prevalence filter of 5% was applied to retain ASVs present in at least 5% of samples within each domain, removing low-prevalence ASVs that disproportionately reflect stochastic amplification or residual contamination in low-biomass samples while preserving the core community signal, consistent with established filtering practices in low-biomass microbiome studies (Karstens et al., 2019).

### 2.6 Statistical analysis

Alpha diversity metrics (Observed richness, Shannon index and Simpson indices) were calculated using the phyloseq package on rarefied datasets. Differences in *α* diversity (Observed richness, Shannon, Simpson) between delivery groups (TV vs. CS) were tested within each gastrointestinal region using two-sided Wilcoxon rank sum tests. *p* values were adjusted across regions using the Benjamini–Hochberg FDR (significance = FDR < 0.05). Benjamini–Hochberg correction was applied separately for each alpha diversity analysis set, including (i) within-index comparisons across regions and (ii) combined analyses across indices.

Beta diversity was assessed by calculating Bray–Curtis dissimilarities on compositional (relative abundance) data within each gastrointestinal region (colon, ileum, jejunum, meconium, and rumen swab), for TV and CS samples. Per-region treatment effects were tested using PERMANOVA (adonis2; vegan v2.6–4; Oksanen et al., 2022) with 9,999 unrestricted permutations. Because each calf contributed a single sample per region, stratification by calf was unnecessary. *p*-values were adjusted across the five regions using Benjamini–Hochberg FDR correction. A combined PERMANOVA across all regions was performed with the model Farm + sample_regions × Treatment (marginal tests; by = ‘margin’; 9,999 permutations) to test interaction effects between gastrointestinal region and delivery method while adjusting for farm. Homogeneity of multivariate dispersion was examined using *betadisper* and *permutest* across region by treatment groups. For ordination, counts were transformed using the centred log ratio (CLR) method (Aitchison, 1982), via the microbiome R package (v1.23.1), in which zero relative-abundance values were replaced with half the minimum non-zero relative abundance observed in the dataset (pseudocount = 5.35 × 10^−5^) prior to log transformation. Euclidean distances (Aitchison) were visualised by principal coordinates analysis (PCoA) and non-metric multidimensional scaling (NMDS).

UniFrac distances (unweighted) were computed based on a maximum likelihood phylogenetic tree constructed with DECIPHER (v2.30.0) and phangorn (v2.11.1) under a GTR + *Γ* + I model. Amplicon sequence variants were aligned over 252 bp after masking positions with gaps or ambiguous bases using *DECIPHER*. The resulting alignment was filtered to remove columns with > 50% gaps, and the final tree was midpoint rooted prior to UniFrac calculation.

Differential abundance analysis at the genus level was performed using MaAsLin2 (v1.22.0) (Mallick et al., 2021). ASV counts were collapsed at the genus level, total sum scaled to relative abundance, and log transformed prior to modelling (corresponding to the ‘TSS’ normalisation and ‘LOG’ transform options in MaAsLin2). Genera were filtered to retain those present in ≥ 10% of samples at a relative abundance ≥ 0.01% before analysis. Delivery method and gastrointestinal region were included as fixed effects, with calf ID included as a random effect to account for the hierarchical structure arising from multiple gastrointestinal regions sampled per animal at the same time point. This combined model approach was preferred over separate per-region analyses as it uses all available data simultaneously and accounts for region effects statistically rather than by subsetting, thereby maximising power for detecting delivery-method associations. Zeros were handled internally by the MaAsLin2 default zero inflated linear model. All models converged normally and no genera were excluded due to model fitting failures.

Multiple testing correction was applied using the Benjamini–Hochberg procedure, and results *q* ≤ 0.05 were considered statistically significant. This threshold was selected to account for the multiple testing burden inherent to high dimensional microbiome datasets, while maintaining sensitivity to biologically meaningful associations.

## 3 Results

### 3.1 Sequence data information

qPCR was used to assess bacterial abundance using the standard curve method and expressed as 16S rRNA gene copy number per microlitre of extracted DNA. ZymoBIOMICS™ mock community DNA served as a positive control, while *Staphylococcus aureus* DNA confirmed assay performance. Each plate included no-template controls (NTCs; H₂O), extraction negatives, and positive controls (Zymo DNA standard and *Staphylococcus aureus* DNA). No-template controls (NTCs) were undetected or amplified late (Ct 32–37), consistent with negligible plate contamination. Extraction negatives were generally late as well, though one ileum batch showed low level background (negatives Ct 27–31). Positive controls amplified as expected (Zymo Ct 7–16; *S. aureus* Ct 6–8) and were used as in plate calibrators.

The chosen 515F/806R primer pair was used, which amplifies both bacterial and archaeal populations Archaeal sequences were detected at very low abundance across all samples. While additional archaeal taxa were initially amplified, these were removed during post-filtering due to relative abundances falling below the 0.01% threshold; Methanobrevibacter was the sole archaeal genus to survive filtering and was subsequently excluded from diversity analyses, which were restricted to bacterial communities.

The 95 samples (including positive and negative controls) produced 8.14 million raw reads of which 3.92 million high-quality non-chimeric reads were retained (48.15%). The average read depth after chimera removal was 41,256 ± 14,402 reads per sample. Stepwise read loss (mean relative to input) was 25.80% during filtering, 29.55% during denoising forward, 29.89% during denoising reverse, 37.77% during merging, and 52.66% following chimera removal. Chimera removal proportions were similar between delivery modes within each region (median ~16–24% across regions; two-sided Wilcoxon rank-sum tests with Benjamini–Hochberg correction: Meconium *q* = 0.11, Jejunum *q* = 0.51, Ileum *q* = 0.90, Colon *q* = 0.57, Rumen swab *q* = 0.74; all *q* > 0.05), indicating no evidence of delivery-mode–specific bias introduced by chimera filtering (Supplementary Figure S2). Potential contaminants were identified using the decontam package with prevalence- and frequency-based models. Extraction negative controls were included across sequencing runs. Negative controls were characterised by low-complexity community profiles dominated by known reagent-associated taxa, consistent with background amplification rather than genuine biological signal, and amplified late in qPCR (Ct 27–31) relative to biological samples. In the ileum extraction batch where low-level background amplification was detected in negative controls (Ct 27–31), the dominant genera *Sporomusa* and *Herbaspirillum* were not flagged by either the prevalence or frequency decontamination models: neither was consistently present in the extraction negatives, and both showed a positive correlation with sample DNA concentration, consistent with genuine biological signal rather than reagent contamination. The proportion of reads retained following decontamination was high and consistent across most sample types: colon (mean 99.6%), ileum (mean 99.8%), and jejunum (mean 96.2%). Meconium and rumen swab samples showed slightly lower mean retention (94.4 and 91.1% respectively), with a small number of individual samples showing substantially higher read removal, reflecting a greater contamination burden in these lower-biomass sample types and indicating that the decontamination pipeline was functioning as intended. Prior to contaminant removal, ordination confirmed that biological samples were clearly separated from negative controls in multivariate space, with mean Bray-Curtis distances of 0.73 and 0.97 between each negative control and the full set of biological samples, supporting the biological integrity of the retained community signal.

In total, 134 ASVs were flagged as contaminants and removed, retaining 94.60% of reads. Following this step, negative and positive controls were excluded from the dataset. One meconium sample (meconium12) had very low sequencing depth (2,496 raw reads; 285 retained after filtering) and failed downstream merging and chimera removal, resulting in zero retained ASVs. This sample was therefore excluded from the dataset, leaving 88 samples for downstream analysis. The final dataset, after the 5% prevalence filter, consisted of 778 ASVs, of which 775 were identified as bacteria and 3 as archaea. Zymo Mock community controls for each of the extractions sample batch were assessed on the post decontam ASV abundance table and were all highly correlated with *r_s_* value ranging from 0.97 to 1.00 (*p* < 0.01).

### 3.2 Alpha diversity

**Figure 1:**
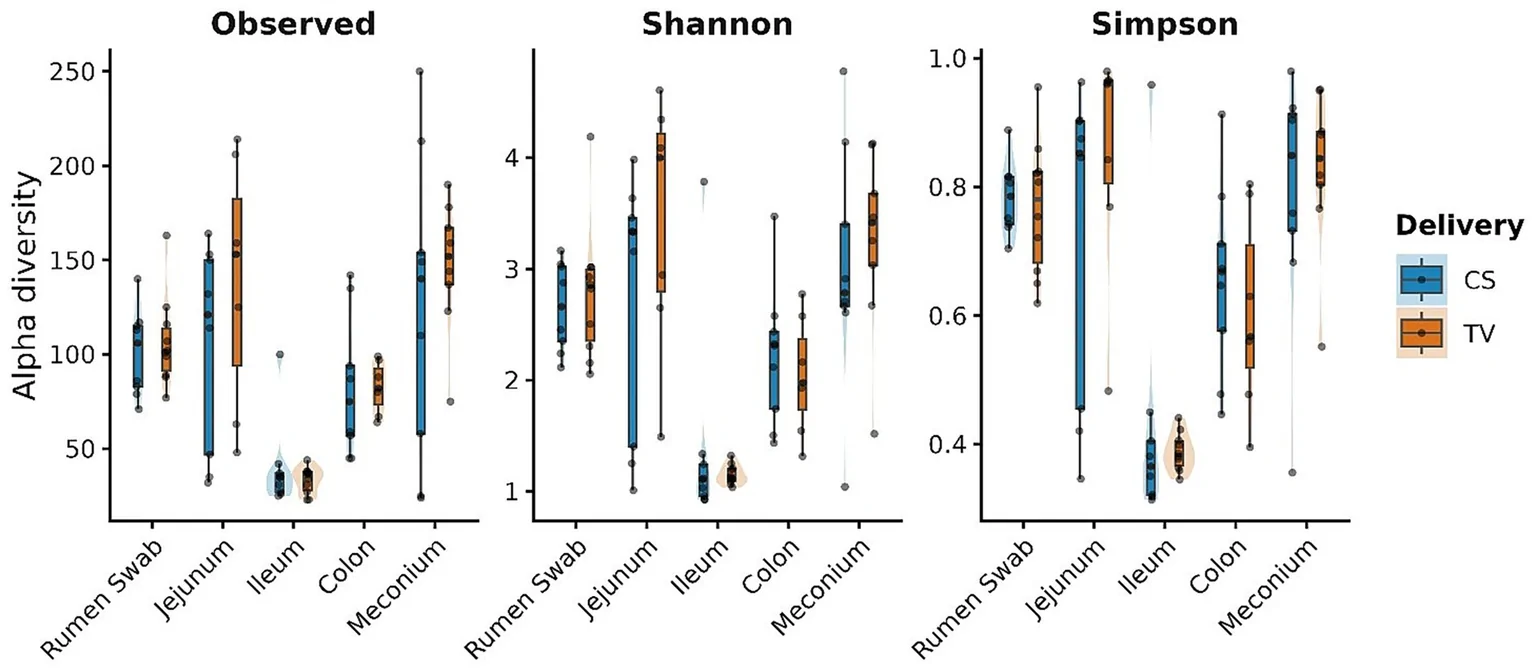
Bacterial alpha diversity across intestinal regions and delivery groups. Boxplots show Observed richness, Shannon, and Simpson indices for calves delivered by caesarean section (CS, blue) and transvaginal delivery (TV, red). Alpha diversity indices were calculated from bacterial ASVs only. Diversity differed significantly among regions (Kruskal–Wallis, *p* < 0.05) but there were no differences between delivery methods within any region (Wilcoxon; BH-adjusted q ≥ 0.74).

Bacterial alpha diversity (Observed, Shannon, and Simpson indices) varied across intestinal region (Kruskal–Wallis, *p* < 0.05, Figure 1), reflecting differences in the microbial complexity along the GIT. The richness and diversity were lowest in the ileum and highest in the jejunum and meconium, while the colon and rumen swabs displayed intermediate values. These regional patterns are consistent with established physiological differences between compartments, including variation in nutrient availability, pH, and oxygen gradients (Donaldson et al., 2016; Steele et al., 2016).

Alpha diversity analyses were restricted to bacterial ASVs, as archaeal taxa were low in abundance and unevenly distributed across regions in neonatal calves, precluding robust cross-region comparisons.

**Figure 2:**
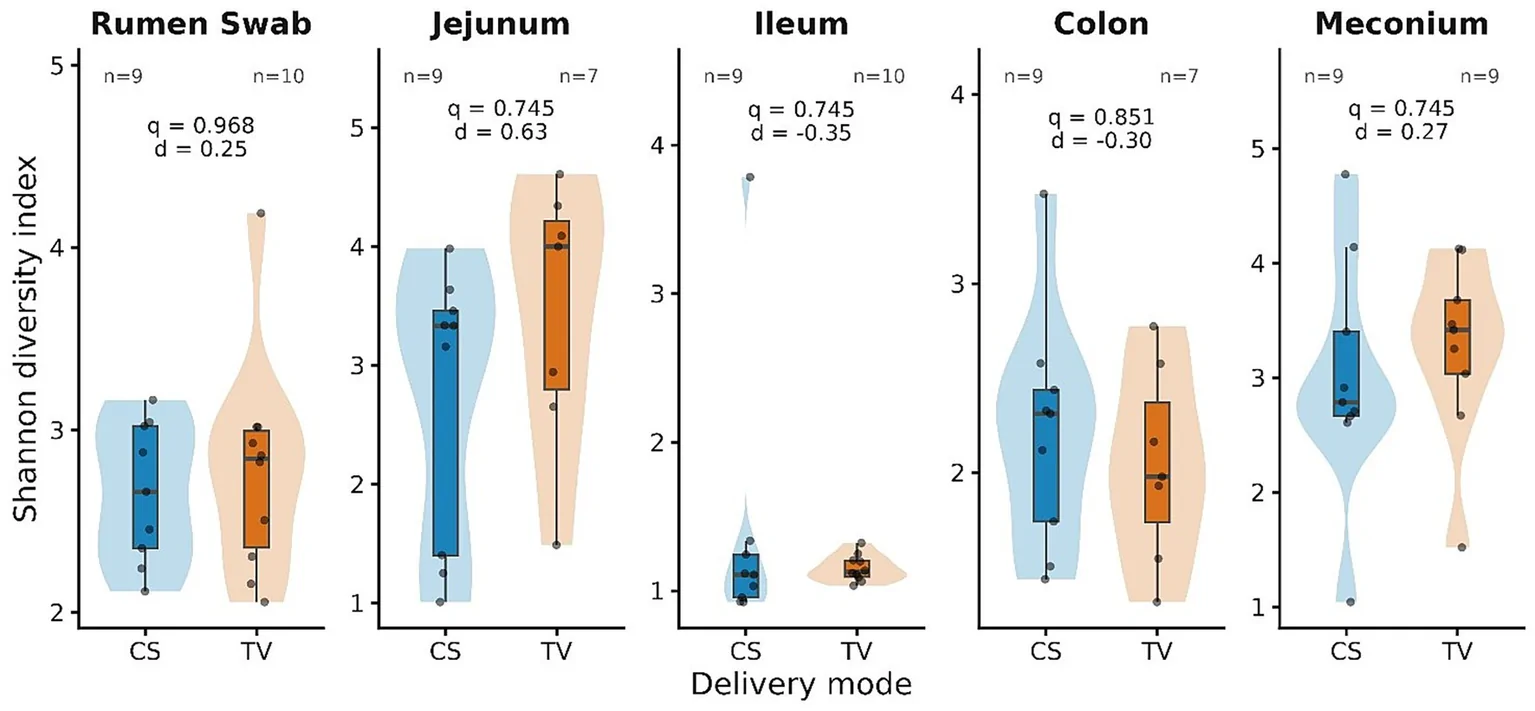
Shannon diversity by delivery methods across GIT regions. Violin–boxplots show genus-level Shannon diversity for calves delivered TV (blue) or by CS (red), faceted by region (Colon, Ileum, Jejunum, Meconium, Rumen Swab). Points are individual samples; boxes indicate median and IQR; violins depict the distribution. Group sizes (n) are printed above each box. Within each facet, the BH-adjusted Wilcoxon *q*-value (*q*) and Cohen’s d (TV–CS) are annotated. No within region differences were significant following FDR correction.

In contrast, there were no within region differences detected between delivery methods for any index (Wilcoxon rank sum, BH-adjusted *q* ≥ 0.74; supplementary Table S2). BH correction was applied separately for (i) Shannon diversity across five regions (Table 1) and (ii) all alpha diversity indices (Observed, Shannon, Simpson) across five regions (Supplementary Table S2). For Shannon diversity, per region distributions and test statistics (BH-adjusted *q* and Cohen’s *d*, TV–CS) are shown in Figure 2. Across all regions, Shannon diversity did not differ by delivery method after multiple testing correction (all *q* ≥ 0.75; Wilcoxon), and effect sizes were small to moderate (|d| ≤ 0.47; Table 1).

**Table 1:** Shannon diversity (genus-level bacteria) by delivery method within gastrointestinal regions.

Region	TV median (IQR)	CS median (IQR)	ΔMedian	*n*	*d*	*P*	*q*
Rumen Swab	2.84 (2.36–3.00)	2.66 (2.35–3.02)	0.18	10/9	0.25	0.97	0.97
Jejunum	4.00 (2.80–4.22)	3.33 (1.40–3.46)	0.67	7/9	0.63	0.17	0.74
Ileum	1.13 (1.10–1.20)	1.11 (0.96–1.24)	0.02	10/9	−0.35	0.45	0.74
Colon	1.98 (1.74–2.37)	2.31 (1.74–2.44)	−0.33	7/9	−0.30	0.68	0.85
Meconium	3.42 (3.04–3.68)	2.79 (2.67–3.40)	0.63	9/9	0.27	0.39	0.74

Values are median (IQR). Δmedian *=* TV – CS. Cohen’s *d* > 0 indicates higher mean in TV. *p* = Wilcoxon rank-sum; *q* = Benjamini–Hochberg FDR across regions. *n =* TV/CS. No comparison was significant after FDR correction (all *q* ≥ 0.74). Regions ordered proximal to distal; meconium listed last.

### 3.3 Beta diversity

**Figure 3:**
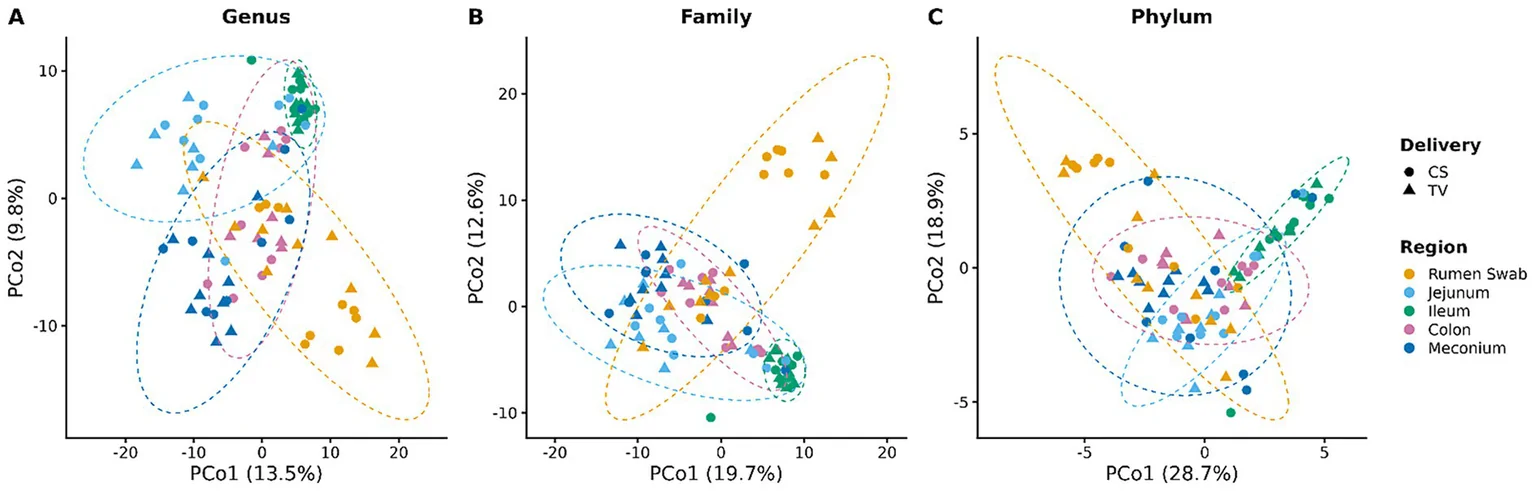
Principal Coordinates Analysis (PCoA) of CLR-transformed microbial communities at genus **(A)**, family **(B)**, and phylum **(C)** levels. Ordinations are based on Euclidean distances (Aitchison geometry). Points represent individual samples coloured by gastrointestinal region and shaped by delivery method (CS, circle; TV, triangle). Dashed ellipses indicate 95% confidence intervals per region. The percentage of variance explained by each axis is shown in parentheses. At all taxonomic levels, samples segregate primarily by anatomical region, with rumen swab and jejunum communities showing the most distinct separation. Variance explained by the first two axes increases from genus (23.3%) to phylum (47.6%), reflecting reduced dimensionality at higher taxonomic ranks.

**Figure 4:**
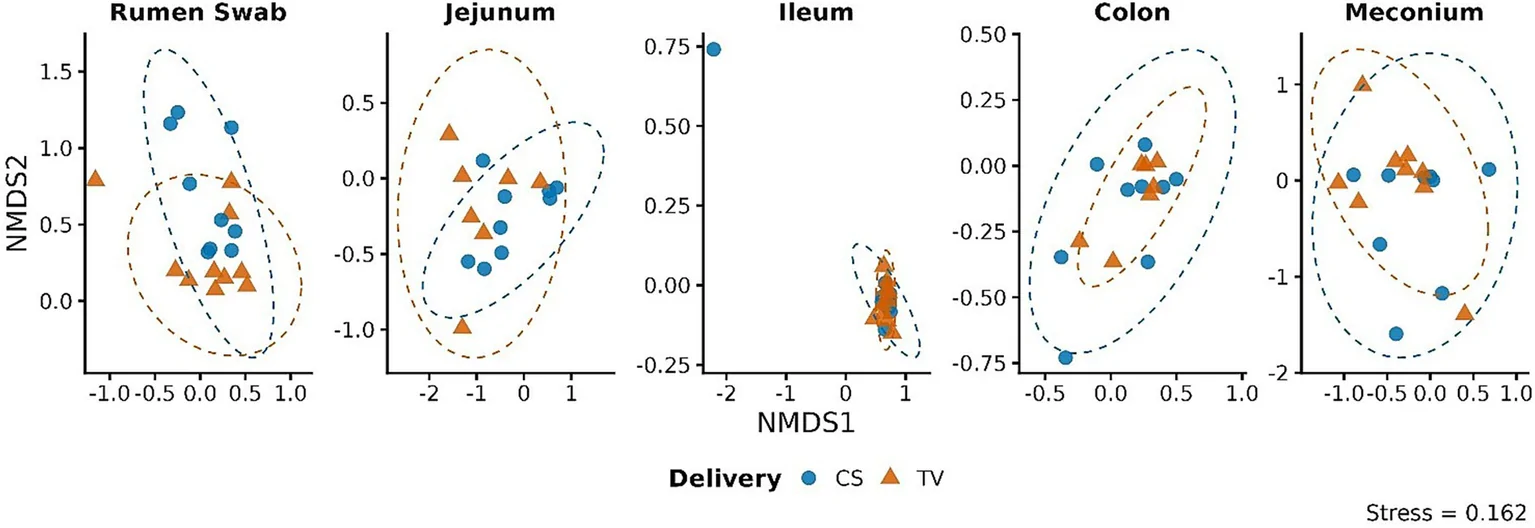
Non-metric multidimensional scaling (NMDS) ordination of Bray–Curtis dissimilarities at the genus level, faceted by gastrointestinal region (ordered proximal to distal; meconium last). Points represent individual samples coloured and shaped by delivery method (CS, circle; TV, triangle). Dashed ellipses indicate 95% confidence intervals per delivery group within each region. Stress = 0.16. The ileum displayed the most compact clustering, reflecting low within-region compositional variability, while the jejunum and colon showed greater sample dispersion. Within all regions, CS and TV ellipses overlap extensively, consistent with the absence of significant delivery- method effects on community composition (PERMANOVA, all *q* > 0.20).

Beta diversity was evaluated at the genus level using Bray–Curtis dissimilarities calculated from relative abundance data. Compositional beta diversity was explored using CLR-transformed abundance data at genus, family, and phylum levels, with Euclidean (Aitchison) distances visualised by PCoA (Figure 3). NMDS ordination of Bray Curtis dissimilarities revealed that microbial community composition was primarily structured by gastrointestinal region, with the ileum and rumen swabs showing the most distinct clustering (Figure 4). Within each region, samples overlapped extensively between caesarean and transvaginal delivered calves, indicating a limited effect of delivery method.

In the combined PERMANOVA across all regions (adonis2; Bray–Curtis; 9,999 permutations; marginal tests), inclusion of Farm as a covariate did not reveal a significant effect (R^2^ = 0.01, *F* = 1.35, *p* = 0.16). Multivariate dispersion also did not differ significantly between farms (PERMDISP: *F* = 0.31, *p* = 0.584), confirming homogeneous community variability across locations. Gastrointestinal region explained a substantial proportion of compositional variation (R^2^ = 0.24, *F* = 6.57, *p* < 0.001), while there was no significant main effect of delivery method (R^2^ = 0.01, *F* = 1.10, *p* = 0.29). A modest Region × Delivery interaction was detected (R^2^ = 0.06, *F* = 1.61, *p* = 0.02), indicating that delivery-associated differences vary slightly across anatomical sites.

Per-region PERMANOVAs were conducted to assess the effect of delivery method within each gastrointestinal region separately (adonis2, Bray–Curtis, 9,999 permutations). Following Benjamini–Hochberg correction across regions, no individual region sampled showed a statistically significant effect of delivery method (colon R^2^ = 0.05, *q* = 0.61; ileum R^2^ = 0.06, *q* = 0.48; jejunum R^2^ = 0.13, *q* = 0.20; meconium R^2^ = 0.06, *q* = 0.48; rumen swab R^2^ = 0.10, *q* = 0.20) (Table 2).

**Table 2:** PERMANOVA (adonis2; 9,999 permutations) of Bray–Curtis dissimilarities from genus-level relative abundance data.

Model	Factor	Df	R^2^	F	*p*	*q*
Per-region	Rumen Swab	1	0.1	1.81	0.08	0.2
	Jejunum	1	0.13	2.04	0.06	0.2
	Ileum	1	0.06	1.08	0.38	0.48
	Colon	1	0.05	0.78	0.61	0.61
	Meconium	1	0.06	1.1	0.34	0.48
Combined	Farm	1	0.01	1.35	0.16	NA
	Sample regions	4	0.24	6.57	<0.001	NA
	Treatment	1	0.01	1.1	0.29	NA
	Region × Treatment	4	0.06	1.61	0.02	NA

Per-region models tested delivery method (TV vs. CS) with unrestricted permutations (one sample per calf per region). The combined model included Farm, Sample region, Treatment, and their interaction as marginal terms. *q* = Benjamini–Hochberg adjusted *p*-value across the five per-region tests.

Multivariate dispersion differed among Region × Treatment groups (PERMDISP; *F* = 6.43, *p* < 0.001) but did not differ between delivery methods within any individual region (all *p* > 0.08), indicating that the per-region PERMANOVA results are not confounded by unequal dispersion. Across all beta diversity analyses, gastrointestinal region was the dominant structuring factor, while delivery method accounted for negligible compositional variance, a pattern consistent across Bray-Curtis, Aitchison, and UniFrac distances.

### 3.4 UniFrac

**Figure 5:**
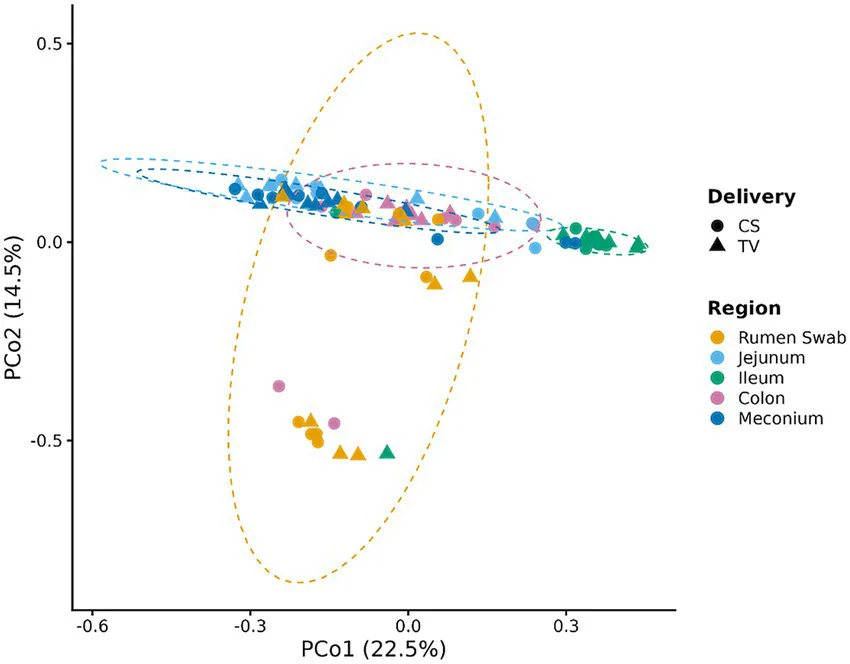
Principal Coordinates Analysis (PCoA) of unweighted UniFrac distances at the ASV level. Points represent individual samples coloured by gastrointestinal region and shaped by delivery method (CS, circle; TV, triangle). Dashed ellipses indicate 95% confidence intervals per region. The first two axes explain 22.5 and 14.5% of total phylogenetic variation, respectively. Samples cluster primarily by anatomical region, with ileum communities forming a tight cluster distinct from other sites and rumen swab samples showing the greatest within-region dispersion. Within each region, CS and TV samples overlap extensively, consistent with the absence of a significant delivery- method effect (PERMANOVA, all *q* > 0.20).

Phylogenetic beta diversity was further examined using unweighted UniFrac distances derived from the maximum likelihood tree of aligned ASVs. Principal Coordinates Analysis (PCoA; Figure 5) revealed clear separation of microbial communities by gastrointestinal region, consistent with Bray–Curtis and CLR/Aitchison analyses. Within each region, caesarean and transvaginal samples overlapped extensively, indicating minimal phylogenetic differentiation attributable to delivery method. PERMANOVA on UniFrac distances confirmed strong regional effects (R^2^ = 0.24, *F* = 6.58, *p* < 0.001) but no significant effect of delivery method (R^2^ = 0.01, *F* = 1.08, *p* = 0.31), with no significant Region × Delivery interaction (R^2^ = 0.03, *F* = 0.92, *p* = 0.63). Per-region PERMANOVAs confirmed no statistically significant delivery method differences within any region following Benjamini–Hochberg correction (all *q* > 0.20).

### 3.5 Taxonomic composition of the GIT microbiota during early life

#### 3.5.1 Phylum

Fifteen bacterial phyla were detected across all regions sampled. Communities were dominated by Proteobacteria (overall mean 67.82%), Firmicutes (16.36%), Bacteroidota (7.79%), and Actinobacteriota (7.43%), which together accounted for over 99% of reads (Supplementary Table S3). Proteobacteria was the most abundant phylum in every region, ranging from 55.81% in the jejunum to 84.48% in the ileum, where it comprised the vast majority of the community. The ileum was the least diverse at phylum level, with Proteobacteria and Firmicutes (10.12%) together exceeding 94% of reads. In contrast, the jejunum harboured the most balanced phylum-level composition, with notable contributions from Bacteroidota (18.14%) alongside Firmicutes (18.28%). Rumen swab communities were distinguished by the highest Firmicutes (22.36%) and Actinobacteriota (10.36%) proportions, while meconium showed the second-highest Actinobacteriota abundance (13.49%). The colon was intermediate, dominated by Proteobacteria (77.02%) with Firmicutes (12.80%) and Bacteroidota (6.06%). Rare phyla (each < 0.1% overall mean RA) included Acidobacteriota, Spirochaetota, Campylobacterota, and Fibrobacterota, although Deinococcota, while low overall (0.25%), was notably enriched in rumen swabs (0.82%). All values represent mean relative abundances across samples within each region.

#### 3.5.2 Genus

Genus-level community profiles were dominated by a small number of taxa. Overall, the most abundant genus was *Sporomusa* (15.95*%),* followed by *Pseudomonas* (7.51%), *Herbaspirillum* (6.46%), *Cutibacterium* (4.81%), *Cupriavidus* (4.46%), and *Staphylococcus* (4.36%).

In the ileum, the community was the most concentrated, with *Sporomusa* (30.71%), *Herbaspirillum* (12.99%), *Pseudomonas* (10.68%), *Cupriavidus* (8.05%), and *Clostridium sensu stricto 3* (6.43%) together accounting for 68.9% of reads. In the colon, the leading genera were *Sporomusa* (18.37%), *Pseudomonas* (7.99%), *Herbaspirillum* (6.58%), *Acinetobacter* (5.90%), and *Cupriavidus* (5.63%) (top 5 sum 44.5%). The jejunum was characterised by *Sporomusa* (8.81%), *Acinetobacter* (7.41%), *Pseudomonas* (5.61%), *Prevotella* (5.59%), and *Herbaspirillum* (4.81%) (32.2%). In meconium, *Pseudomonas* (8.89%) was the most abundant genus, followed by *Sporomusa* (7.66%), *Acinetobacter* (5.18%), *Corynebacterium* (4.73%), and *Cutibacterium* (4.53%) (31.0%). Rumen swabs were enriched for *Sporomusa* (13.01%), *Staphylococcus* (12.32%), *Methylobacterium*–*Methylorubrum* (9.90%), *Cutibacterium* (7.64%), and *Pseudomonas* (4.21%) (47.1%).

**Figure 6:**
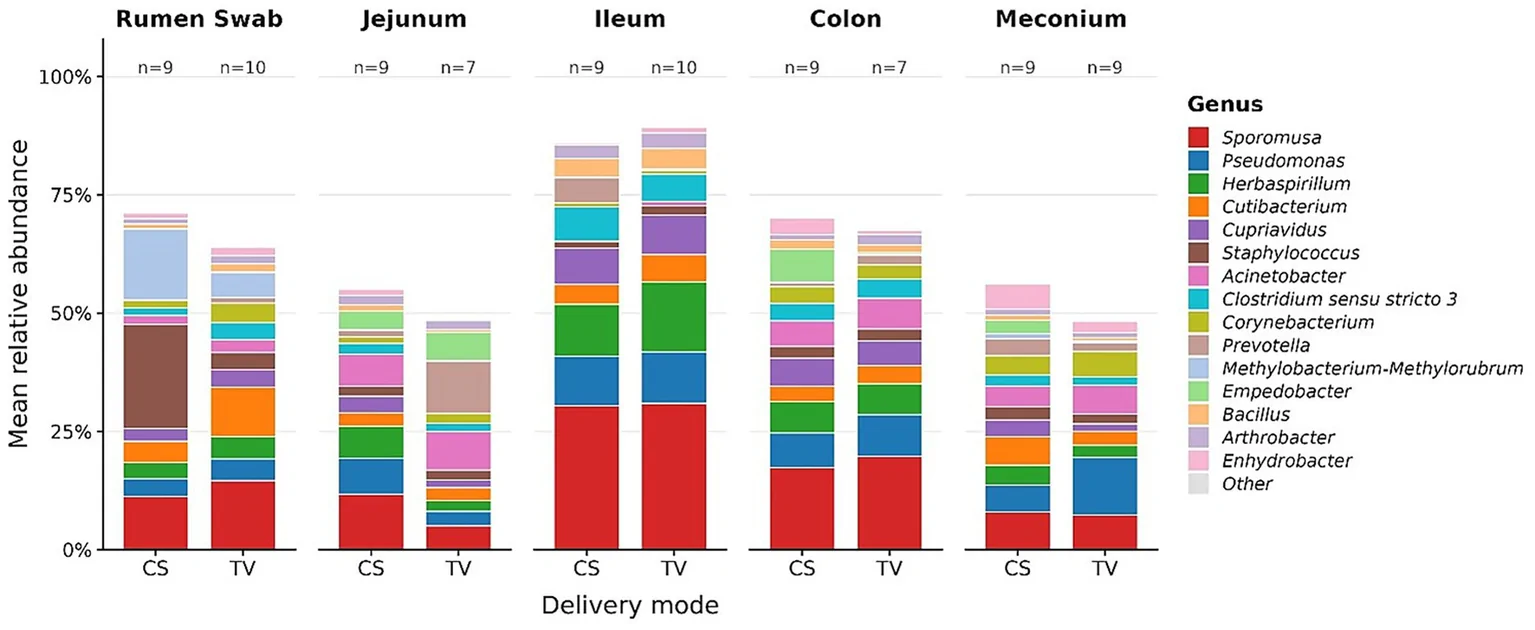
Mean relative abundance of the top 15 bacterial genera by delivery method within each gastrointestinal region. Stacked bars represent the mean genus-level relative abundance for calves delivered by caesarean section (CS) or transvaginal delivery (TV) within each region. Genera are ranked by overall mean abundance; taxa outside the top 15 are grouped as “Other” (grey). Sample sizes (*n*) are indicated above each bar. Genus-level composition was dominated by *Sporomusa*, *Pseudomonas*, and *Herbaspirillum* across most regions, with region-specific enrichments (e.g., *Methylobacterium–Methylorubrum* in rumen swabs, *Cutibacterium* in rumen swabs and jejunum).

These patterns are characterised by high relative abundances of *Sporomusa* together with facultative aerobes and environmentally associated taxa such as *Pseudomonas*, *Acinetobacter*, *Herbaspirillum*, *Cupriavidus*, and *Cutibacterium* (Alipour et al., 2018; Song et al., 2018). The dominance of Proteobacteria and aerotolerant genera across all regions is consistent with the expected pioneer colonisation phase in neonatal calves. The relative abundance profiles of the top 15 bacterial genera varied markedly by gastrointestinal region (Figure 6). Colon and ileum samples were dominated by *Sporomusa*, *Pseudomonas*, and Herbaspirillum, whereas meconium samples showed a more even distribution led by *Pseudomonas*, *Sporomusa*, and *Acinetobacter*. Jejunum communities were characterised by *Sporomusa*, *Acinetobacter*, *Pseudomonas*, and *Prevotella*, while rumen swab samples were enriched for *Sporomusa*, *Staphylococcus*, *Methylobacterium*–*Methylorubrum*, and *Cutibacterium*. Within each region, the overall community composition was broadly similar between calves delivered by TV and CS.

### 3.6 Differential abundance analysis

To identify bacterial genera that differed in relative abundance across intestinal regions and delivery mode, multivariable MaAsLin2 models were applied to genus-level abundance data (TSS-normalised and log-transformed). The fixed effects included region and delivery method (CS vs. TV), while cow ID was included as a random effect to account for repeated measures. The ileum was set as the reference level for region comparisons. After false discovery rate (FDR) correction, 191 genus–metadata associations were detected at *q* ≤ 0.05. Anatomical region was the strongest predictor of genus abundance, accounting for nearly all significant associations (189/191). Compared with the ileum (model reference), distinct region-specific bacterial signatures were observed.

The meconium displayed the highest number of differentially abundant genera (*n =* 66, *q* ≤ 0.05). Genera enriched in the meconium relative to the ileum included *Sphingomonas*, *Streptococcus*, *Corynebacterium*, *Paenibacillus*, *Oscillospiraceae UCG-005*, and *Acinetobacter* (q < 0.001), while *Herbaspirillum* and *Bacillus* were depleted (*q* < 0.001). The jejunum also exhibited extensive genus-level differentiation (*n =* 56, *q* ≤ 0.05). Genera enriched in the jejunum included *Prevotella_7*, *Alloprevotella*, *Bacteroides*, *Faecalibacterium*, and *Prevotella* (*q* < 0.001), as well as *Sharpea* and *Psychrobacter* (*q* < 0.001). The rumen swab contained 39 region-associated genera. Strong enrichments were observed for *Methylobacterium*–*Methylorubrum*, *Actinomycetospora*, *Deinococcus*, *Sphingomonas*, *Corynebacterium*, and *Hymenobacter* (all *q* < 0.001). In the colon, 28 genera differed significantly in relative abundance compared with the ileum. Enrichments were detected for *Acinetobacter*, *Paenibacillus*, *Sphingomonas*, *Corynebacterium*, and *Prevotella* (*q* < 0.001), with *Finegoldia* (*q* < 0.001) and *Peptoniphilus* (*q* < 0.001) also showing increased abundance.

When comparing calves delivered by caesarean section (CS) or transvaginally (TV), two genera remained significant after FDR correction. *Paraclostridium* [reclassified from *Paeniclostridium*; (Sasi Jyothsna et al., 2016)] was more abundant in TV-delivered calves (q = 0.003). *Pseudoxanthomonas* showed a similar pattern (*q* = 0.047). Intestinal region was the dominant factor shaping genus-level microbial composition (189/191 significant associations), while only two genera were significantly associated with delivery method.

### 3.7 Farm specific genus composition

PERMANOVA of Bray–Curtis dissimilarities at the genus level indicated no significant effect of Farm on community composition (R^2^ = 0.01, *F* = 1.26, *p* = 0.20). In contrast, Sample region accounted for a substantially larger proportion of variation (R^2^ = 0.24, *F* = 6.56, *p* < 0.001), explaining approximately 24% of total compositional variance.

Tests of multivariate dispersion (betadisper; 9,999 permutations) revealed no difference in dispersion between farms (*F* = 0.31, *p* = 0.584), indicating homogeneous variability across farms. However, dispersion differed significantly among sample regions (*F* = 14.78, p < 0.001), suggesting that regional differences in community composition reflect a combination of centroid separation and dispersion heterogeneity (see Supplementary Table S4 for full PERMANOVA and PERMDISP results).

## 4 Discussion

This study used a purposefully designed animal model to provide a controlled comparison of birth delivery method on initial GIT microbial colonisation. All calves were sired by a single Aberdeen Angus bull, born to first-calving heifers of similar breed type, and maintained under equivalent dietary and health management within each farm. Calves were euthanised within minutes of birth, before colostrum ingestion, contact with the dam, or substantive environmental exposure, thereby isolating the effects of birth delivery route from the postnatal confounding factors that complicate interpretation in human and animal studies (Surlis et al., 2017; O'Hara et al., 2020). Aseptic sampling protocols and multi-layer contamination controls substantially reduced the potential for technical artefact; however, residual contamination cannot be completely excluded in low-biomass samples, and interpretation therefore prioritises convergent, ecologically coherent patterns across samples rather than isolated detections.

The evidence from our systematic study suggests that microbial assembly in the immediate period after birth was shaped primarily by the local ecology of each gastrointestinal region rather than by delivery method. Across five neonatal calf sites sampled under aseptic conditions (rumen swab, jejunum, ileum, colon, and meconium), alpha diversity and community composition varied markedly by intestinal compartment, consistent with strong physiological and biochemical differences between these regions (Malmuthuge et al., 2014; Song et al., 2018). The rumen, jejunum, ileum, colon each present distinct physicochemical conditions in oxygen availability, pH, substrate gradients, epithelial secretions, and developmental maturity (Donaldson et al., 2016; Steele et al., 2016), which create distinct ecological filters that strongly structure initial microbial colonisation. These site-specific selective pressures appear to have outweighed any delivery-associated seeding effects within the present sampling window, with compositional differences between delivery groups remaining small relative to regional variation across all analyses conducted. This finding aligns with observations from controlled human, rodent, and bovine studies (Rutayisire et al., 2016; Zhang et al., 2021a) and refines expectations from the human and rodent literature by demonstrating that site-specific ecology outweighs delivery method at birth in neonatal calves under controlled perinatal conditions (Du et al., 2023).

This strong regional structuring aligns with known functional differences between compartments: the neonatal rumen remains relatively aerobic and undeveloped (Jost et al., 2012) favouring transient or environmentally derived taxa, whereas the ileum and colon are increasingly anaerobic and nutrient-rich, selecting for early fermentative communities (Malmuthuge et al., 2015; Virgínio Júnior and Bittar, 2021).

In the combined PERMANOVA model, region explained 24% of compositional variance, while delivery method accounted for only 1% further supporting the conclusion that intrinsic microenvironmental conditions exert a far stronger influence than birth-related inoculum (Reyman et al., 2019; Shao et al., 2019; Zhang et al., 2021b). The modest Region × Delivery interaction detected in the combined PERMANOVA, despite the absence of significant per-region effects after multiple-testing correction, is most plausibly explained by the aggregation of weak and inconsistent region-specific trends across the five sites sampled. When pooled, these sub-threshold signals produced a detectable interaction term, but their inconsistency across regions and small individual effect sizes preclude attribution to any specific anatomical site and are not consistent with a biologically meaningful or reproducible delivery- method effect. Notably, as we had planned farm of origin explained negligible compositional variance despite the two facilities being geographically distinct and managed independently, suggesting that the patterns reported here are not location site-specific and are likely generalisable across comparable beef production systems.

Differential abundance testing highlighted how local gastrointestinal environments impose distinct selective pressures on early microbial colonisation (Malmuthuge et al., 2015; Zhang et al., 2021a). Each of the regions sampled showed genus level signatures that reflected their underlying physiology, developmental state, and metabolic landscape.

Consistent with its relatively higher oxygen tension, rapid nutrient flux, and exposure to simple carbohydrates in early life, the jejunum showed enrichment of genera including *Prevotella*, *Bacteroides*, and *Faecalibacterium* (Steele et al., 2016). These taxa are broadly associated with carbohydrate metabolism and facultative anaerobic growth in other contexts, consistent with the relatively oxygenated jejunal microenvironment (Sommer and Bäckhed, 2013; Donaldson et al., 2016; Yeoman et al., 2018). However, functional activity cannot be directly inferred from 16S rRNA amplicon data, and these ecological interpretations remain hypotheses to be tested by metagenomic or metabolomic approaches.

In contrast, meconium samples were dominated by taxa characteristic of sparse, early-life biological matrices, including *Sphingomonas*, *Streptococcus*, *Corynebacterium*, and *Paenibacillus*, which are frequently detected in low-biomass neonatal and environmental samples rather than representing stable gut-derived communities. This profile is characteristic of low-biomass matrices containing transient environmental or maternal skin-associated taxa rather than stable gut-derived communities, (Lauder et al., 2016; Eisenhofer et al., 2019; Klein-Jöbstl et al., 2019), consistent with limited in utero microbial exposure and minimal substrate availability in neonatal meconium (Guzman et al., 2020). Rumen swabs were characterised by enrichment of *Methylobacterium–Methylorubrum*, *Sharpea*, and *Corynebacterium*, reflecting the immature, oxygenated rumen, that initially favours oxygen tolerant, facultative or environmentally derived taxa before the rumen develops a fully anaerobic fermentative microbiome (Steele et al., 2016; Amin and Seifert, 2021). By contrast, the colon displayed elevated abundances of *Paenibacillus*, *Prevotella*, and *Finegoldia*, consistent with the establishment of an increasingly anaerobic, carbohydrate fermenting community as hindgut fermentation capacity and mucosal maturation progress during the neonatal period (Song et al., 2018; Zhu et al., 2021; Du et al., 2023).

Collectively, these region-specific signatures 189 of 191 significant genus–metadata associations attributed to anatomical site — exemplify the dominant role of local ecological filtering during initial colonisation (Sommer and Bäckhed, 2013; Malmuthuge et al., 2015; Donaldson et al., 2016). Such strong compartmental filtering readily outweighs transient peripartum inoculation events, which explains why region effects were consistently stronger than those of delivery method (Yeoman et al., 2018; Klein-Jöbstl et al., 2019).

In the immediate perinatal window, microbial communities are characteristically composed of oxygen tolerant pioneers and environmentally sourced taxa (Song et al., 2018; Malmuthuge et al., 2019a). This Proteobacteria-dominant, anaerobe-poor profile is consistent with published neonatal calf datasets sampled within hours of birth. Alipour et al. (2018) reported Proteobacteria as the dominant phylum in rumen and intestinal contents of calves within the first 24 h of life, with obligately anaerobic Firmicutes and Bacteroidota rising in relative abundance only during the first weeks postnatally as gut redox conditions shift. Song et al. (2018) similarly described aerotolerant genera as the predominant early colonisers of the neonatal bovine GIT prior to maturation of a strictly anaerobic community. The low abundance of mature anaerobic taxa in the present dataset is therefore an expected feature of sampling at this developmental stage rather than an anomaly and is fully consistent with the oxygen-consumption model of gut ecological succession. Consistent with this expectation, all regions were dominated by Proteobacteria and aerotolerant genera such as *Sporomusa*, *Pseudomonas*, *Herbaspirillum*, *Cupriavidus*, *Acinetobacter*, and *Cutibacterium*. *Sporomusa* predominated in the ileum and colon, whereas *Pseudomonas* was especially abundant in meconium and ileum; *Staphylococcus, Cutibacterium, and Methylobacterium–Methylorubrum* were most enriched in rumen swabs (Alipour et al., 2018; Song et al., 2018).

The dominance of *Sporomusa*, particularly in the ileum and colon, is notable given its role as an acetogenic bacterium that synthesises acetate from CO₂ and H₂ via the Wood–Ljungdahl pathway (Möller et al., 1984; Drake et al., 2008). Although acetogens are classically regarded as strict anaerobes, *Sporomusa* species exhibit comparatively high oxygen tolerance, maintaining acetate production under low-oxygen conditions through the activity of peroxidase and NADH oxidase rather than catalase or superoxide dismutase (Karnholz et al., 2002; Boga and Brune, 2003). This aerotolerance, albeit at an energetic cost, may confer a selective advantage in the partially oxygenated neonatal gut lumen. Furthermore, *Sporomusa* species can form commensal relationships with aerotolerant, oxygen-consuming microbes that scavenge local oxygen, thereby creating a protected niche for acetogenesis (Bae et al., 2024). This trophic interaction is consistent with the co-dominance of *Sporomusa* alongside oxygen-tolerant Proteobacteria such as *Pseudomonas* and *Herbaspirillum* observed across regions in the present study. The acetate generated via the Wood–Ljungdahl pathway may additionally serve as a carbon and energy source for subsequent colonisers, positioning *Sporomusa* as a metabolically significant pioneer in early gastrointestinal assembly. It should be noted that these functional interpretations are based on the established literature for *Sporomusa* and are not directly demonstrated by the 16S rRNA amplicon data reported here; metagenomic and metabolomic approaches will be required to confirm whether these metabolic activities are operative in the neonatal bovine gut.

These patterns are consistent with ecological models of early life gut assembly, in which facultative aerobes act as pioneer colonisers that consume residual oxygen, thereby conditioning the gastrointestinal environment for the subsequent establishment of strict anaerobes (Sommer and Bäckhed, 2013; Houghteling and Walker, 2015; Donaldson et al., 2016). Variation among gastrointestinal regions is likely driven by site specific redox conditions, substrate availability, and differences in epithelial maturation (Malmuthuge et al., 2015; Zhang et al., 2021b; Du et al., 2023), suggesting that the trajectory of microbial succession from aerotolerant pioneers to obligate anaerobes may proceed at different rates across gastrointestinal compartments.

Delivery method had a negligible impact at birth, with only two genera showing significant differential abundance, *Paraclostridium* and *Pseudoxanthomonas*. *Paraclostridium* is both metabolically versatile and a recognised component of the normal vaginal microbiota in cattle, and its modest enrichment in transvaginally delivered calves is consistent with transient maternal inoculation rather than stable ecological establishment (Kwon et al., 2021; Sim et al., 2022). This association should not, however, be interpreted as evidence of a functional colonisation role; its biological significance remains uncertain given the small sample size, the large number of taxa examined, and the absence of any corresponding community-level differences.

Calves delivered transvaginally traversed the birth canal and were transiently exposed to the dam’s vaginal and faecal microbiota, whereas calves delivered by caesarean section bypassed this route entirely (Alipour et al., 2018; Klein-Jöbstl et al., 2019). Vaginal delivery is also associated with physiological stress responses, including elevated cortisol, transient respiratory acidosis, and activation of acute-phase pathways, which may influence mucosal immune function and, by extension, early microbial colonisation (Surlis et al., 2017). The prostaglandin-based induction protocol used here deliberately avoided exogenous corticosteroids to prevent confounding effects on the neonatal microbiota. It should be noted, however, that delivery method and parturition were not fully decoupled in this design. Given the necessity for planned veterinary intervention TV calves experienced induced labour and its associated physiological sequelae, whereas CS calves were delivered surgically prior to the full physiological processes of vaginal birth. Observed differences between groups therefore reflect the combined effect of birth route and parturition induction rather than microbial exposure during canal passage alone. Despite these physiological and immunological distinctions, the near-absence of delivery-associated differences in microbial composition suggests that the sampling window, within minutes of birth, preceded any substantive microbial establishment attributable to birth-route exposure (Malmuthuge et al., 2015; Yeoman et al., 2018). Whether delivery-induced differences in mucosal immune signalling subsequently influence microbial succession over the hours and days following birth remains an important question for longitudinal investigation.

*Pseudoxanthomonas*, an environmental Gammaproteobacterium typically associated with soil and water, was similarly enriched in TV-delivered calves. Although not a canonical member of the bovine vaginal microbiota, the maternal vaginal tract is not a sterile environment and can harbour environmentally derived taxa through contact with faeces, skin, and bedding. The enrichment of *Pseudoxanthomonas* specifically in TV-delivered calves, sampled within 25 min of birth, before colostrum ingestion or substantive environmental contact, is consistent with exposure during passage through the birth canal, although postnatal environmental contribution cannot be fully excluded. Both genera thus represent transient signals of birth-route exposure that are biologically plausible given the timing of sampling and the low biomass of the neonatal gut but are unlikely to reflect sustained delivery-method effects. Instead, these findings reinforce the dominance of postnatal ecological selection over birth-associated microbial exposure (Yeoman et al., 2018; Klein-Jöbstl et al., 2019).

This pattern differs from human studies, where caesarean delivery often produces pronounced early microbial alterations, including reduced *Bacteroides* colonisation and increased facultative anaerobes. These differences highlight species specific variation in physiology, perinatal management, and environmental exposure that modulate the extent to which delivery method shapes gut colonisation (Palmer et al., 2007; Biasucci et al., 2008; Long et al., 2021; Inchingolo et al., 2024). Comparable findings have been reported in ruminant studies, where early microbial communities show minimal differentiation by delivery method (Alipour et al., 2018; Yeoman et al., 2018; Klein-Jöbstl et al., 2019). The small effect sizes, together with the limited number of delivery-associated genera after multiple testing correction (2 of 191 significant associations), indicate that birth route is a weak determinant of microbial assembly in the immediate postnatal period. Across all complementary analyses, gastrointestinal region consistently emerged as the dominant structuring factor, while delivery method explained approximately 1% of community variation and was associated with significant differential abundance in only two genera after FDR correction. Collectively, these results suggest that, within the present dataset, delivery method exerts at most a modest and regionally dependent influence on gastrointestinal microbial composition at birth, though subtle or transient effects below the detection threshold of this study cannot be excluded. These patterns are more consistent with a limited biological effect than lack of statistical sensitivity and support the conclusion that initial microbial establishment in calves is governed predominantly by local postnatal ecological conditions rather than the maternal inoculation route (Alipour et al., 2018).

The narrow sampling window, within 25 min of birth, preceded colostrum ingestion or substantive environmental contact, thereby severely limiting opportunities for sustained maternal microbial transfer. Under such conditions, any inoculum acquired during vaginal delivery is likely to be minimal, transient, and rapidly superseded by strong compartment specific selective pressures (Yeoman et al., 2018). Stringent aseptic handling minimised environmental contamination that typically amplify delivery method differences in human cohorts, including skin contact, ambient microbiota and early feeding, thereby isolating the intrinsic biological effects of delivery method (Rutayisire et al., 2016; Reyman et al., 2019).

This experimental design provides a stringent test of delivery method effects; if birth route were a major determinant of microbial establishment at this timepoint, differences would be expected to be detectable even under these controlled conditions. The near-absence of such differences, with only two of 191 statistically significant associations attributable to delivery method, is consistent with any initial delivery-associated inoculum being outweighed by compartment-specific ecological conditions within the present sampling window, though effects that are subtle, transient, or below the detection threshold of this study cannot be excluded (Meale et al., 2016; O'Hara et al., 2020).

Several detected genera are known to occur as contaminants in low-biomass microbiome datasets. Extensive safeguards were therefore implemented in the current study following best-practice guidelines (Salter et al., 2014; Lauder et al., 2016; de Goffau et al., 2018; Eisenhofer et al., 2019; Karstens et al., 2019), including extraction negatives, mock community standards, and qPCR-informed decontamination. The decontamination workflow, which combined prevalence- and qPCR-informed frequency models, removed ASVs consistent with reagent or low-biomass contamination while retaining the vast majority of reads.

The dominant genera *Pseudomonas, Acinetobacter, Cutibacterium, Methylobacterium-Methylorubrum, Cupriavidus, Herbaspirillum*, and *Sphingomonas* have previously been reported as reagent- or environment-associated contaminants in low-biomass microbiome studies (Salter et al., 2014; de Goffau et al., 2018; Eisenhofer et al., 2019). To evaluate this directly, we examined our two extraction-negative (kit-only) controls. One control contained reads of all seven taxa (0.19–7.18% of its reads); the second contained none, consistent with an isolated, batch-specific contamination event rather than reagent contamination across the study. For four of the seven taxa *(Herbaspirillum, Pseudomonas, Cutibacterium*, and *Cupriavidus*), the proportion detected in this control was similar in magnitude to their mean relative abundance in biological samples. All seven genera were nonetheless detected at near-universal prevalence across the 88 biological samples (48–88 of 88), spanning multiple calves, farms, and gastrointestinal regions, a distribution not explained by a single extraction event, and none of the corresponding ASVs were flagged as contaminants by our decontam workflow. A contribution from reagent-associated background cannot be fully excluded. Accordingly, we do not assign strong biological significance to the abundance of individual genera from this list, and interpretations in this study are anchored to patterns that were reproducible across multiple samples and regions, supported by ecological coherence with the redox and developmental state of each compartment, and consistent with the broader pioneer colonisation literature. Isolated or low-abundance detections of these taxa were not over-interpreted.

Furthermore, genus-level characterisation of cattle farm air microbiomes has identified Bacillus, Lysinibacillus, Corynebacterium, and Streptomyces among prominent airborne taxa across farm settings (Guo et al., 2025), a profile substantively distinct from the genera detected across GIT regions in the present study; while this distinction does not exclude reagent or contact contamination, it reduces the likelihood that the dominant regional signal reflects airborne environmental input specifically.

These findings may be considered in the context of the ongoing debate on prenatal versus postnatal microbial colonisation, though this study was not designed to directly test the sterile womb hypothesis. The immediate post birth sampling design, stringent asepsis collection, multi-layer negative controls, and prevalence based decontamination approach support the interpretation that substantial microbial colonisation occurs primarily during and after birth (Lauder et al., 2016; Pérez-Muñoz et al., 2017; de Goffau et al., 2018). The detection of a single archaeal taxon, Methanobrevibacter, at very low abundance and restricted distribution is more consistent with incidental or early-stage exposure than with the establishment of a mature, obligate anaerobic community *in utero*, however, given that methanogens are strict anaerobes, no inference regarding oxygen tolerance is intended or appropriate here (Steele et al., 2016; Song et al., 2018; Du et al., 2023).

This interpretation aligns with recent evidence suggesting minimal or transient microbial exposure *in utero* (Khan et al., 2015; de Goffau et al., 2019; Theis et al., 2019) and that many reported signals likely reflect environmental or reagent contamination rather than true colonisation (Pérez-Muñoz et al., 2017). Nonetheless, sporadic reports of bacterial DNA or viable cells in foetal and placental samples indicate that limited prenatal microbial exposure cannot be fully excluded (Guzman et al., 2020; Campisciano et al., 2021; Mizrahi et al., 2021; Dera et al., 2024). Taken together, the observations presented here are consistent with a model of minimal or transient prenatal exposure, followed by significant microbial establishment during the immediate postnatal period.

Within the immediate postnatal window sampled here, perinatal management and local compartmental ecology appear to outweigh delivery method in shaping the earliest detectable microbiota (Meale et al., 2016; Klein-Jöbstl et al., 2019; Virgínio Júnior and Bittar, 2021). If this pattern persists beyond the immediate birth window, postnatal interventions, through colostrum management, hygiene practices, and diet, may represent more tractable targets than delivery route for steering early microbial colonisation toward beneficial communities (Meale et al., 2016; Malmuthuge and Guan, 2017; Virgínio Júnior and Bittar, 2021), however, this remains to be tested in longitudinal studies that evaluate microbial persistence and colonisation success beyond the timepoint examined here. The nominal treatment signal observed in rumen swabs, although not statistically robust, suggests the rumen as a potentially sensitive site for detecting early ecological perturbations and may inform future targeted intervention strategies (Steele et al., 2016; Amin and Seifert, 2021).

The cross-sectional design, capturing a single timepoint at birth, precludes characterisation of temporal microbial succession. Whether the neonatal GIT is sterile or colonised at birth remains the central interpretive question, and longitudinal data from the same animal model document a decline in aerotolerant taxa within the first weeks of life, with more mature anaerobic communities becoming established in the rumen and colon thereafter (O'Hara et al., 2020; Stafford et al., 2025), a trajectory consistent with genuine postnatal colonisation rather than artefactual signal, though direct longitudinal tracking from birth across timepoints remains necessary to confirm this. Future studies should therefore extend this approach across multiple GIT regions to determine whether the modest delivery-associated signals observed here become amplified, persist, or dissipate with environmental exposure and feeding. Library depths were adequate for genus-level comparisons but limit confidence in resolving rare taxa, and shotgun metagenomics or metaproteomics and local oxygen or redox profiling will be essential to connect taxonomic shifts to ecosystem function and metabolic activity (Schären et al., 2017; Arshad et al., 2021). Despite the use of stringent controls, low biomass contamination cannot be fully excluded, and interpretation accordingly prioritised reproducible multi-sample patterns, effect sizes, and ecological plausibility rather than isolated or low-abundance detections (Lauder et al., 2016; de Goffau et al., 2018; Eisenhofer et al., 2019). Functional readouts (metabolites such as SCFAs, redox markers) and host responses (mucosal transcripts, IgA/IgG) were not assayed here. These will be crucial for future determination of whether small compositional differences at birth translate into meaningful ecosystem function or calf health outcomes (Malmuthuge et al., 2019b; Mizrahi et al., 2021). Host integration, such as epithelial transcriptomics, mucosal immunology or metabolite mapping, would provide mechanistic insight into region structured pioneer communities and barrier and immune maturation (Khan et al., 2015; Malmuthuge et al., 2015; Guzman et al., 2020). Integrating these approaches within multi-omic frameworks will be crucial to resolving how microbial succession and host physiology co-evolve during the early life of ruminants (Ma et al., 2020; Mizrahi et al., 2021).

These findings should also be interpreted in the context of the experimental power. In our original study design, submitted for ethical approval we hypothesised a detectable difference in genera of approximately 30% (80% power, *α* = 0.05) in diversity or gene expression as a consequence of birth delivery method, in accordance with the variance observed for our previously published rumen and intestinal digesta microbiome studies (O'Hara et al., 2020; Stafford et al., 2025). Additionally, reduced sample sizes within specific regions, particularly the jejunum (TV *n =* 7, CS *n =* 9) and colon (TV *n =* 7, CS *n =* 9), limit the statistical power of the within-region PERMANOVA and differential abundance analyses, and subtle delivery-method effects within individual compartments cannot be formally excluded. The observed effect sizes were small (Shannon Cohen’s d ≤ 0.63; per-region PERMANOVA R^2^ ≤ 0.13), well below this detection threshold. Accordingly, the detection of only two delivery-associated genera among 191 significant associations is consistent with the minimal effect of birth route reflecting genuine biological similarity between transvaginal and caesarean-delivered calves at birth, rather than insufficient statistical power, though subtle effects below the detection threshold of this study cannot be excluded (Husso et al., 2021). Within the constraints of the present study, this is consistent with regional ecological conditions, rather than delivery route, being the dominant determinant of microbial community assembly immediately post birth (Malmuthuge et al., 2015; Zhang et al., 2021b).

The possibility of type II error warrants consideration given the variable sample sizes across gastrointestinal regions and the inter-individual variability inherent to microbiome datasets. However, several aspects of the data argue against insufficient statistical power as the primary explanation for the null delivery-method results. The jejunum, which had the fewest TV samples and represents the most proximal sampled intestinal site, returned the largest per-region effect size of any individual region yet remained non-significant after correction, the opposite pattern to what would be expected if low sample size were masking a true effect. Effect sizes were consistently small across all analyses and well below the difference the study was powered to detect. The absence of delivery-method effects was also consistent across alpha diversity, beta diversity, and differential abundance analyses, reducing the likelihood of a systematic false negative. Taken together, these observations are more consistent with a genuinely limited biological effect of delivery method at this timepoint than with underpowering, though effects smaller than those anticipated in the *a priori* power calculation cannot be formally excluded.

Overall, the results support a model in which the perinatal calf microbiome is a region structured, oxygen tolerant pioneer community in which delivery method exerts, at most, a minimal influence at birth. Through the combination of stringent low biomass decontamination, ecologically anchored interpretation, and effect size centred inference, this work contributes a reproducible characterisation of the immediate post-birth ruminant microbiota under controlled perinatal conditions, though persistence, colonisation success, and metabolic activity remain to be evaluated in longitudinal studies. These findings delineate clear priorities for future research, particularly longitudinal, functional, and host integrated investigations that can determine whether early microbial differences influence subsequent gut development, metabolic function, and calf health or performance.

## 5 Conclusion

In conclusion, gastrointestinal region was the dominant driver of microbial community structure detectable within 25 min of birth, whereas delivery method exerted only a minor influence under controlled perinatal conditions at this timepoint. These findings provide a rigorously controlled characterisation of the immediate post-birth ruminant microbiota and suggest that, at the moment of sampling, community composition was more strongly associated with anatomical site than with maternal inoculation route; whether this reflects longer-term colonisation dynamics remains to be determined by longitudinal investigation. Importantly, our inferences are restricted to the immediate postnatal period, and whether delivery method contributes to later divergence remains unknown. The results nonetheless highlight the importance of postnatal management factors in shaping microbial trajectories and emphasise the need for longitudinal and functional studies to determine whether early microbial differences influence subsequent gut development, immune maturation or calf health. Together, these insights refine our understanding of pioneer microbial communities in neonatal calves and point toward targeted, postnatal strategies to support optimal microbiome development.

## Data Availability

The data analyzed in this study can be found in the NCBI (https://www.ncbi.nlm.nih.gov) under BioProject accession PRJNA1466506. Additional supplementary data can be accessed through Open Science Framework at: https://doi.org/10.17605/OSF.IO/28UEZ.

## Glossary

ASV: amplicon sequence variant
AVMA: American Veterinary Medical Association
BH: Benjamini-Hochberg
CLR: centred log ratio
CS: caesarean section
Ct: cycle threshold
DADA2: Divisive Amplicon Denoising Algorithm 2
DAFM: Department of Agriculture Food and Marine
FDR: false discovery rate
GIT: gastrointestinal tract
GTR: general time reversible
HPRA: Health Products Regulatory Association
IQR: interquartile range
MaAsLin2: Multivariable Association discovery in population-scale meta-omics Studies
MC: mock community
NMDS: non-metric multidimensional scaling
NTC: no-template control
PCoA: principal coordinates analysis
PERMANOVA: permutational multivariate analysis of variance
PERMDISP: permutational analysis of multivariate dispersion
qPCR: quantitative polymerase chain reaction
RA: relative abundance
SCFA: short-chain fatty acid
TAEC: Teagasc Animal Ethics Committee
TSS: total sum scaling
TV: transvaginal delivery
