# Supplementary material for: Microbial colonisation of the neonatal beef calf gastrointestinal tract: limited influence of birth delivery method across five anatomical regions
Source: Front Microbiol. 2026 Jul 29;17:1883207. doi: 10.3389/fmicb.2026.1883207 (PMC13492196; doi:10.3389/fmicb.2026.1883207)
Supplement: Supplementary file 1 [file Data_Sheet_1.docx]

Supplementary Material

# 1 Supplementary Figures and Tables

For more information on Supplementary Material and for details on the different file types accepted, please see [here](https://www.frontiersin.org/guidelines/author-guidelines#supplementary-material).

## 1.1 Supplementary Figures

Supplementary Table S1. Overview of calves and gastrointestinal sites sampled at birth.
Calves were delivered either by caesarean section (CS) or transvaginal delivery (TV) across four experimental replicates and two farms. Binary values indicate whether a sample was collected from each anatomical site (1 = sample collected; 0 = no sample available).

| Calf Sequence | Treatment | Gender | Replicate | Farm | Colon | Ileum | Jejunum | Meconium | Rumen Swab |
| --- | --- | --- | --- | --- | --- | --- | --- | --- | --- |
| Calf_1 | CS | FEMALE | 1 | 1 | 1 | 0 | 1 | 1 | 1 |
| Calf_2 | CS | MALE | 1 | 1 | 1 | 1 | 0 | 0 | 0 |
| Calf_3 | TV | MALE | 1 | 1 | 1 | 1 | 0 | 0 | 0 |
| Calf_4 | TV | FEMALE | 1 | 1 | 0 | 0 | 0 | 1 | 0 |
| Calf_5 | CS | FEMALE | 2 | 1 | 0 | 1 | 1 | 1 | 1 |
| Calf_6 | CS | HEIFER | 2 | 1 | 1 | 1 | 1 | 1 | 1 |
| Calf_7 | CS | FEMALE | 2 | 1 | 1 | 1 | 1 | 1 | 1 |
| Calf_8 | CS | MALE | 2 | 1 | 1 | 1 | 1 | 1 | 1 |
| Calf_9 | TV | FEMALE | 2 | 1 | 1 | 1 | 0 | 1 | 1 |
| Calf_10 | TV | MALE | 2 | 1 | 1 | 1 | 1 | 1 | 1 |
| Calf_11 | TV | FEMALE | 2 | 1 | 1 | 1 | 1 | 1 | 1 |
| Calf_12 | TV | MALE | 2 | 1 | 0 | 1 | 1 | 1 | 1 |
| Calf_13 | CS | FEMALE | 3 | 2 | 1 | 1 | 1 | 1 | 1 |
| Calf_14 | CS | MALE | 3 | 2 | 1 | 1 | 1 | 1 | 1 |
| Calf_15 | TV | FEMALE | 3 | 2 | 1 | 1 | 0 | 1 | 1 |
| Calf_16 | TV | FEMALE | 3 | 2 | 0 | 0 | 0 | 1 | 1 |
| Calf_17 | TV | MALE | 3 | 2 | 1 | 1 | 1 | 1 | 1 |
| Calf_18 | TV | MALE | 3 | 2 | 0 | 1 | 1 | 1 | 1 |
| Calf_19 | CS | FEMALE | 4 | 2 | 1 | 1 | 1 | 1 | 1 |
| Calf_20 | CS | MALE | 4 | 2 | 1 | 1 | 1 | 1 | 1 |
| Calf_21 | TV | MALE | 4 | 2 | 0 | 1 | 1 | 0 | 1 |
| Calf_22 | TV | FEMALE | 4 | 2 | 1 | 1 | 1 | 1 | 1 |

**Supplementary Table S2.** **Alpha diversity by delivery method within each gastrointestinal region (Wilcoxon rank-sum tests).** Wilcoxon rank-sum tests comparing Observed richness, Shannon, and Simpson indices between calves delivered by caesarean section (CS) and transvaginal delivery (TV) within each intestinal region. Regions ordered proximal to distal; meconium listed last. W = Wilcoxon test statistic; *P* = unadjusted Wilcoxon *P*-value; *q* = Benjamini–Hochberg adjusted P-value across all regions and indices. ns = not significant at *q* > 0.05. Alpha diversity was estimated on rarefied data (depth = 1,612 reads; seed = 42).

| Region | Index | n (TV) | n (CS) | Median TV | IQR TV | Median CS | IQR CS | *W* | *P* | *q* | Sig. |
| --- | --- | --- | --- | --- | --- | --- | --- | --- | --- | --- | --- |
| Rumen Swab | Observed | 10 | 9 | 101.50 | 22.25 | 106.00 | 32.00 | 40.00 | 0.72 | 0.98 | ns |
|  | Shannon | 10 | 9 | 2.84 | 0.64 | 2.66 | 0.67 | 46.00 | 0.97 | 1.00 | ns |
|  | Simpson | 10 | 9 | 0.78 | 0.14 | 0.79 | 0.07 | 46.00 | 0.97 | 1.00 | ns |
| Jejunum | Observed | 7 | 9 | 153.00 | 88.50 | 121.00 | 103.00 | 18.50 | 0.19 | 0.88 | ns |
|  | Shannon | 7 | 9 | 4.00 | 1.42 | 3.33 | 2.06 | 18.00 | 0.17 | 0.88 | ns |
|  | Simpson | 7 | 9 | 0.96 | 0.16 | 0.85 | 0.45 | 19.00 | 0.21 | 0.88 | ns |
| Ileum | Observed | 10 | 9 | 35.50 | 9.75 | 35.00 | 10.00 | 45.00 | 1.00 | 1.00 | ns |
|  | Shannon | 10 | 9 | 1.13 | 0.11 | 1.11 | 0.29 | 35.00 | 0.45 | 0.88 | ns |
|  | Simpson | 10 | 9 | 0.38 | 0.04 | 0.37 | 0.08 | 36.00 | 0.50 | 0.88 | ns |
| Colon | Observed | 7 | 9 | 82.00 | 19.00 | 75.00 | 37.00 | 25.00 | 0.53 | 0.88 | ns |
|  | Shannon | 7 | 9 | 1.98 | 0.63 | 2.31 | 0.70 | 36.00 | 0.68 | 0.98 | ns |
|  | Simpson | 7 | 9 | 0.57 | 0.19 | 0.67 | 0.13 | 39.00 | 0.47 | 0.88 | ns |
| Meconium | Observed | 9 | 9 | 152.00 | 30.00 | 140.00 | 96.00 | 31.00 | 0.44 | 0.88 | ns |
|  | Shannon | 9 | 9 | 3.42 | 0.64 | 2.79 | 0.74 | 30.00 | 0.39 | 0.88 | ns |
|  | Simpson | 9 | 9 | 0.84 | 0.08 | 0.85 | 0.18 | 38.00 | 0.86 | 1.00 | ns |

Supplementary Table S3. Mean relative abundance (%) of bacterial phyla across gastrointestinal regions. Values represent the mean relative abundance calculated across all samples within each region for calves delivered by caesarean section and transvaginal delivery combined. Phyla are ordered by descending overall mean abundance. *n* per region: Rumen Swab = 19, Jejunum = 16, Ileum = 19, Colon = 16, Meconium = 18.

| Phylum | Rumen Swab | Jejunum | Ileum | Colon | Meconium | Overall |
| --- | --- | --- | --- | --- | --- | --- |
| Proteobacteria | 61.66 | 55.81 | 84.48 | 77.02 | 59.22 | 67.82 |
| Firmicutes | 22.36 | 18.28 | 10.12 | 12.8 | 18.09 | 16.36 |
| Bacteroidota | 4.44 | 18.14 | 3.17 | 6.06 | 8.52 | 7.79 |
| Actinobacteriota | 10.36 | 7.08 | 2.03 | 3.91 | 13.49 | 7.43 |
| Deinococcota | 0.82 | 0.03 | 0 | 0.12 | 0.21 | 0.25 |
| Fusobacteriota | 0 | 0.53 | 0 | 0 | 0.02 | 0.1 |
| Patescibacteria | 0.08 | 0.05 | 0.05 | 0.01 | 0.11 | 0.06 |
| Abditibacteriota | 0.16 | 0 | 0 | 0 | 0.07 | 0.05 |
| Verrucomicrobiota | 0.02 | 0.02 | 0.11 | 0.03 | 0.08 | 0.05 |
| Cyanobacteria | 0.02 | 0.01 | 0 | 0.04 | 0.07 | 0.03 |
| Acidobacteriota | 0.02 | 0.01 | 0.03 | 0.02 | 0.01 | 0.02 |
| Desulfobacterota | 0.05 | 0 | 0 | 0 | 0.03 | 0.02 |
| Fibrobacterota | 0 | 0.01 | 0.01 | 0 | 0.06 | 0.02 |
| Campylobacterota | 0.02 | 0.03 | 0 | 0 | 0 | 0.01 |
| Spirochaetota | 0 | 0 | 0 | 0 | 0.02 | 0.01 |

**Supplementary Table S4. PERMANOVA and homogeneity of multivariate dispersion (PERMDISP) for Farm and Sample region on genus-level Bray–Curtis dissimilarities.** PERMANOVA (adonis2; 9,999 permutations; marginal tests) tested the effects of Farm and Sample region on Bray–Curtis dissimilarities calculated from genus-level relative abundance data. PERMDISP (betadisper + permutest; 9,999 permutations) assessed homogeneity of multivariate dispersion for each factor separately. Df = degrees of freedom; Sum Sq = sum of squares; Mean Sq = mean squares; *F* = F statistic; *p* = permutation P-value.

| Analysis | Factor | Df | Sum Sq | Mean Sq | *F* | N.Perm | *P* |
| --- | --- | --- | --- | --- | --- | --- | --- |
| PERMANOVA | Farm | 1 | 0.172 | 0.172 | 1.26 | 9,999 | 0.202 |
|  | Sample region | 4 | 3.599 | 0.900 | 6.56 |  | < 0.001 |
|  | Residual | 82 | 11.239 | 0.137 |  |  |  |
| PERMDISP | Farm | 1 | 0.015 | 0.015 | 0.31 | 9,999 | 0.589 |
|  | Residuals | 86 | 4.171 | 0.049 |  |  |  |
| PERMDISP | Sample region | 4 | 1.479 | 0.370 | 14.78 | 9,999 | < 0.001 |
|  | Residuals | 83 | 2.077 | 0.025 |  |  |  |

Supplementary Table S5. Alpha diversity sensitivity analysis at rarefaction depth 5,000 reads per sample. Wilcoxon rank-sum tests comparing delivery method (CS vs TV) within each gastrointestinal region for Observed richness, Shannon, and Simpson indices. Analysis restricted to samples retaining >= 5,000 reads following quality filtering and decontamination (n = 76 of 88 samples). *q* = Benjamini-Hochberg FDR adjusted p-value across all 15 region-metric comparisons. Regions ordered proximal to distal; meconium listed last. No comparison was significant after FDR correction (all *q* = 1.00).

| Region | Measure | n (CS) | n (TV) | Median CS | IQR CS | Median TV | IQR TV | W statistic | P value | q value (BH) |
| --- | --- | --- | --- | --- | --- | --- | --- | --- | --- | --- |
| Rumen swab | Observed | 9 | 10 | 114 | 43 | 115 | 27.5 | 38.5 | 0.624 | 1 |
| Rumen swab | Shannon | 9 | 10 | 2.635 | 0.632 | 2.884 | 0.625 | 45 | 1 | 1 |
| Rumen swab | Simpson | 9 | 10 | 0.791 | 0.068 | 0.781 | 0.144 | 45 | 1 | 1 |
| Colon | Observed | 8 | 7 | 89.5 | 51.5 | 93 | 23.5 | 24.5 | 0.728 | 1 |
| Colon | Shannon | 8 | 7 | 2.407 | 0.383 | 2.015 | 0.634 | 36 | 0.397 | 1 |
| Colon | Simpson | 8 | 7 | 0.69 | 0.082 | 0.574 | 0.191 | 38 | 0.281 | 1 |
| Ileum | Observed | 5 | 6 | 42 | 10 | 42 | 4.25 | 17.5 | 0.714 | 1 |
| Ileum | Shannon | 5 | 6 | 1.162 | 0.358 | 1.148 | 0.136 | 16 | 0.931 | 1 |
| Ileum | Simpson | 5 | 6 | 0.38 | 0.11 | 0.376 | 0.045 | 16 | 0.931 | 1 |
| Jejunum | Observed | 8 | 7 | 139 | 65 | 177 | 103.5 | 18.5 | 0.297 | 1 |
| Jejunum | Shannon | 8 | 7 | 3.395 | 0.815 | 4.125 | 1.452 | 19 | 0.336 | 1 |
| Jejunum | Simpson | 8 | 7 | 0.861 | 0.151 | 0.964 | 0.154 | 19 | 0.336 | 1 |
| Meconium | Observed | 7 | 9 | 172 | 62.5 | 167 | 28 | 30 | 0.916 | 1 |
| Meconium | Shannon | 7 | 9 | 2.941 | 1.053 | 3.476 | 0.773 | 26 | 0.606 | 1 |
| Meconium | Simpson | 7 | 9 | 0.847 | 0.169 | 0.844 | 0.1 | 31 | 1 | 1 |

Supplementary Figure S1. Rarefaction of alpha diversity across preprocessing stages. Rarefaction curves for each sample showing Observed ASV richness and Shannon diversity as a function of sequencing depth. Panels: (A) Pre-decontamination (controls included), (B) Post-decontamination (controls included), (C) Post-filtering (controls removed).

Supplementary Figure S2. Chimera removal proportions by delivery method across gastrointestinal regions. Boxplots show the percentage of merged reads removed as chimeras for calves delivered by caesarean section (CS, blue) or transvaginal delivery (TV, orange) within each region, ordered proximal to distal (meconium last). Points represent individual samples. Sample sizes are indicated above each panel. Benjamini–Hochberg adjusted *q*-values from within-region Wilcoxon rank-sum tests are annotated per facet (Rumen Swab *q* = 0.745; Jejunum *q* = 0.51; Ileum *q* = 0.90; Colon *q* = 0.57; Meconium *q* = 0.12). No region showed a significant difference in chimera removal between delivery methods after multiple testing correction (all *q* > 0.05). Median chimera removal ranged from approximately 16–24% across regions.
